# A comparison of cecal colonization of *Salmonella enterica *serotype Typhimurium in white leghorn chicks and *Salmonella*-resistant mice

**DOI:** 10.1186/1471-2180-8-182

**Published:** 2008-10-16

**Authors:** Christine P Sivula, Lydia M Bogomolnaya, Helene L Andrews-Polymenis

**Affiliations:** 1 Department of Veterinary Pathobiology, College of Veterinary Medicine, Texas A&M University, College Station, TX, USA; 2 Department of Microbial and Molecular Pathogenesis, College of Medicine, Texas A&M University System Health Science Center, College Station, TX, USA; 3 Research Animal Resources, University of Minnesota, Minneapolis, MN, USA

## Abstract

**Background:**

Salmonellosis is one of the most important bacterial food borne illnesses worldwide. A major source of infection for humans is consumption of chicken or egg products that have been contaminated with *Salmonella enterica *serotype Typhimurium, however our knowledge regarding colonization and persistence factors in the chicken is small.

**Results:**

We compared intestinal and systemic colonization of 1-week-old White Leghorn chicks and *Salmonella*-resistant CBA/J mice during infection with *Salmonella enterica *serotype Typhimurium ATCC14028, one of the most commonly studied isolates. We also studied the distribution of wild type serotype Typhimurium ATCC14028 and an isogenic *invA *mutant during competitive infection in the cecum of 1-week-old White Leghorn chicks and 8-week-old CBA/J mice. We found that although the systemic levels of serotype Typhimurium in both infected animal models are low, infected mice have significant splenomegaly beginning at 15 days post infection. In the intestinal tract itself, the cecal contents are the major site for recovery of serotype Typhimurium in the cecum of 1-week-old chicks and *Salmonella*-resistant mice. Additionally we show that only a small minority of *Salmonellae *are intracellular in the cecal epithelium of both infected animal models, and while SPI-1 is important for successful infection in the murine model, it is important for association with the cecal epithelium of 1-week-old chicks. Finally, we show that in chicks infected with serotype Typhimurium at 1 week of age, the level of fecal shedding of this organism does not reflect the level of cecal colonization as it does in murine models.

**Conclusion:**

In our study, we highlight important differences in systemic and intestinal colonization levels between chick and murine serotype Typhimurium infections, and provide evidence that suggests that the role of SPI-1 may not be the same during colonization of both animal models.

## Background

There are an estimated 1.4 million cases annually of food related illness caused by non-typhoidal *Salmonella *in the United States [1]. *S*. Typhimurium, a non-typhoidal serotype of subspecies I *Salmonella enterica (subspecies Enterica)*, causes enteric disease in a wide range of species including humans, cattle, and swine [2]. In humans, *S*. Typhimurium usually causes clinical disease limited to the gastrointestinal tract that is characterized by diarrhea, abdominal cramps and fever. However, non-typhoidal *Salmonella *infections can be fatal in young, elderly and immunocompromised persons [3]. Serotype Typhimurium is the most prevalent serotype associated with food related illness in the United States [4-6], and consumption of contaminated poultry and egg products are often associated with clinical illness in humans [5]. In chicks over a few days of age, serotype Typhimurium colonizes the gastrointestinal tract but does not cause clinical disease [7]. Infected chicks are able to shed serotype Typhimurium into the environment for extended periods of time increasing the risk for environmental contamination, spread of the organism within the flock and contamination of the food supply.

The ability of serotype Typhimurium and other non-typhoidal species to cause enteric disease lies partially in their ability to invade the intestinal epithelial cells of the host [8]. Following oral ingestion of serotype Typhimurium, these bacteria adhere to and invade the intestinal epithelial cells and cells of intestinal lymphoid follicles or Peyer's patches [9]. Injection of effector proteins of the *Salmonella *Pathogenicity Island-1 (SPI-1) encoded type III secretion system (TTSS) into intestinal epithelial cells leads to cystoskeletal rearrangement in the host cell that result in the formation of membrane ruffles that engulf the bacteria. Interaction between *Salmonella *and host epithelial cells leads to the recruitment of macrophages and polymorphonuclear leukocytes to the infected intestine [10]. *Salmonella *serotypes that cause enteritis induce fluid secretion into the intestinal lumen leading to an inflammatory diarrhea that is mediated by the SPI-1 TTSS [11-15].

The vast majority of studies of the molecular basis for non-typhoidal *Salmonella *virulence and intestinal colonization have been performed using murine models of infection and *in vitro *cell culture systems. *Salmonella*-susceptible mice, such as BALB/c strains, develop a fatal systemic disease similar to that of Typhoid fever in humans [16,17]. *Salmonella*-resistant mice, including CBA and 129Sv strains, are resistant to development of systemic disease following infection with serotype Typhimurium but instead are asymptomatically colonized [18]. The factors necessary for serotype Typhimurium to colonize livestock models that are natural hosts of *Salmonella *and vectors for this organism into the human food supply are less well defined.

The study of sub-clinical carriage of serotype Typhimurium in poultry is especially important, as a whole different combination of colonization factors may be important to survive the gastrointestinal tract of the asymptomatic chicken than are required for symptomatic colonization of other hosts. Research utilizing the chick model has included pathophysiology of infection in poultry as well as identification of colonization factors [19-25]. However, previous work with poultry has not utilized the serotype Typhimurium isolates which are best studied *in vitro *and in murine models, ATCC14028 and SL1344, making direct comparison of results previously obtained in the chick model with the large amount of data obtained *in vitro *and in murine models over the last 30 years challenging. ATCC14028 is currently undergoing complete sequencing (McClelland, unpublished data), while the sequence of SL1344 is complete and publicly available [26].

In this study we show a direct comparison between intestinal and systemic colonization in chick and *Salmonella*-resistant mouse models, utilizing the well-studied *Salmonella enterica *serotype Typhimurium isolate ATCC14028. We determined the degree of systemic and enteric colonization by wild type ATCC14028 in single infection, and we quantify the distribution of this isolate and an otherwise isogenic SPI-1 mutant (Δ*invA*) during competitive infection in the ceca of 1-week-old White Leghorn chicks and 8-week-old *Salmonella*-resistant CBA/J mice. In addition, we assessed the degree of fecal shedding of serotype Typhimurium ATCC14028 in both chick and murine models of infection.

## Results

### Systemic colonization by *S*. Typhimurium

**Figure 1:**
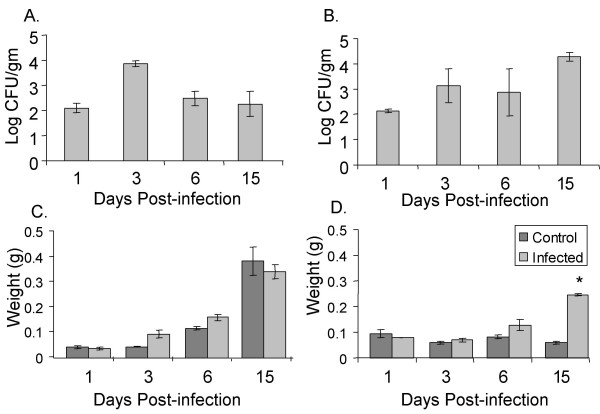
**Systemic colonization following serotype Typhimurium infection in chicks and mice**. Recovery of serotype Typhimurium from the spleen following oral inoculation in chicks (A) and mice (B) is shown as mean log CFU/gm of organ tissue. Mean spleen weight of chicks (C) and mice (D) is shown in grams of organ tissue. Error bars indicate standard error and asterisks indicate a significant difference between control and infected groups at *P *< 0.05.

We determined the level of systemic colonization by serotype Typhimurium by evaluating colonization of the spleen in infected chicks and in infected *Salmonella*-resistant mice over 15 days of infection (Figure 1A, B). In both chicks and mice, the spleen was only lightly colonized after oral infection throughout the experiment at fewer that 10^4 ^organisms per gram of tissue, consistent with the asymptomatic nature of these infections (Figure 1A for Chick, Figure 1B for Mice). We also assayed the colonization of the liver in serotype Typhimurium-infected chicks, which followed the same pattern of colonization with very low numbers of organisms as the spleen in infected chicks (data not shown). We noted a spike in colonization of the systemic organs in chicks at 3 days post infection that was rapidly resolved. Serotype Typhimurium infected CBA/J mice were initially colonized in systemic organs at a very low level, and became progressively colonized with higher numbers of organisms throughout the experiment with a delay relative to infected chicks (Figure 1B). The delay in higher levels of colonization of the systemic organs in mice relative to chicks may be a result organisms using different routes of dissemination in the two animal models, and/or of a delay of organisms passing through the mesenteric lymph node in mice, a structure that birds normally lack [27,28].

We also evaluated the level of systemic infection in both murine and chick models by quantifying splenic and hepatic enlargement in infected animals and in age-matched, uninfected control animals. While the spleen weights of both control and infected groups of chicks increased over time to due rapid growth of these animals, we found that the spleens of infected chicks were not significantly enlarged relative to those of uninfected age-matched chicks (Figure 1C). Similarly, infected chicks did not have hepatic enlargement relative to uninfected control animals (data not shown). These findings are consistent with the very low level of systemic colonization we observed in infected chicks (Figure 1A).

In contrast, we observed a significant splenic enlargement in serotype Typhimurium infected CBA/J mice relative to uninfected age-matched control animals at 15 days post infection (Figure 1D), at a time when bacterial numbers in this organ are increasing, and have surpassed 10^4 ^CFU per gram of tissue (Figure 1B). Infected CBA/J mice also have significant hepatic enlargement at this time point (data not shown). Both splenic and hepatic enlargement in these serotype Typhimurium infected mice continues to 30 days post infection (data not shown). Such enlargement of systemic organs in infected murine models is consistent with the higher level of colonization of these organs by serotype Typhimurium that we observed at 14 days post infection (Figure 1B).

### Dynamics of *S*. Typhimurium intestinal colonization

**Figure 2:**
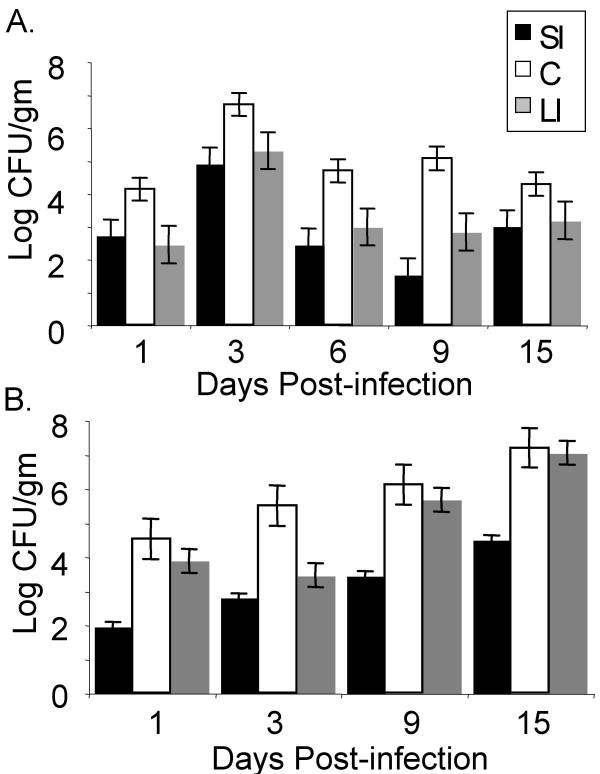
**Intestinal colonization following serotype Typhimurium infection in chicks and mice**. Recovery of serotype Typhimurium from the small intestine (SI), cecum (C), and large intestine (LI) following oral inoculation in chicks (A) and mice (B) is shown as mean log CFU/gm of organ tissue, and error bars denote standard error.

We analyzed colonization of serotype Typhimurium in the small intestine (SI), cecum (C), and large intestine (LI) of infected chicks and infected *Salmonella*-resistant CBA/J mice (Figure 2). Due to the limited availability of SPF chicks, we used one-week old chicks that are reported to be somewhat resistant to serotype Typhimurium infection due to their maturing intestinal microbiota, among other factors [19,29,30]. In both animal models, the ceca were the most heavily colonized organ at all time points post infection (Figure 2), consistent with earlier work [31]. In 1-week-old chicks, the ceca were most heavily colonized with serotype Typhimurium until 3 days post infection, and from this time on serotype Typhimurium was found in the ceca in lower numbers (Figure 2A). The colonization of the small intestine and large intestine in the chick followed similar dynamics to cecal colonization in this model in that the number of organisms in these niches initially increased until day 3 post infection and then decreased over the remainder of the experiment (Figure 2A).

In *Salmonella*-resistant mice however, the level serotype Typhimurium colonization in the cecum steadily increased throughout the duration of the experiment (Figure 2B). Colonization of the small and large intestine in CBA/J mice also increased throughout the duration of the experiment (Figure 2B) in contrast to the chick, with colonization of the large intestine approaching the level of cecal colonization at later points of infection (9 and 15 days post infection). Colonization of the murine small intestine also increased throughout the duration of the experiment but remained at relatively low levels, and was perhaps responsible for continuous seeding of the cecum in mice.

### The influence of SPI-1 in cecal colonization

In order to determine the importance of utilization of the intracellular niche by serotype Typhimurium in cecal colonization we studied the distribution of wild type serotype Typhimurium versus a mutant with a nonfunctional SPI-1 encoded type three secretion system-1. Δ*invA *mutants produce a defective type III secretion system (TTSS-1 encoded on SPI-1) and they are unable to secrete the effectors of this system. Although Δ*invA *mutants adhere to cultured mammalian epithelial cells they are unable to invade these cells, a process thought to be important for intestinal colonization and enteropathogenesis [8,32,33]. We utilized a competitive infection of wild type serotype Typhimurium ATCC14028s and an isogenic Δ*invA *mutant to analyze the distribution of both strains in the whole cecum, cecal contents and cecal epithelium (both cell-associated and intracellular) in both chicks and *Salmonella*-resistant CBA/J mice.

**Figure 3:**
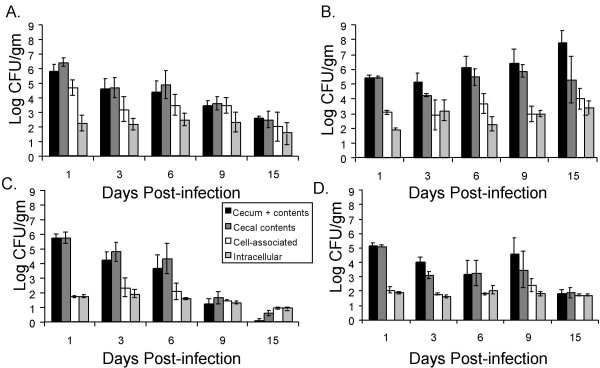
**Different niches for cecal colonization of serotype Typhimurium following oral infection in chicks and mice**. Recovery of wild type ATCC14028 and Δ*invA *mutant from the whole cecum (tissue + contents), the cecal contents only, the cecal epithelium associated (extracellular attached+intracellular), and intracellular to the cecal epithelium of both chicks and mice. Colonization levels of these compartments by WT ATCC14028 in chicks (A) and mice (B), and Δ*invA *mutant of chicks (C) and mice (D) following oral inoculation is shown as mean log CFU/gm of cecal tissue, and error bars denote standard error.

On day 1 post-infection, the ceca of both animal models were heavily colonized with approximately 10^6 ^CFU/gm and 10^5 ^CFU/gm of the wild type ATCC14028 primarily in the cecal contents (Figure 3). The Δ*invA *mutant initially colonized the cecum and its contents in nearly identical numbers to the isogenic wild type serotype Typhimurium in both animal models (Figure 3C, D). The number of cell-associated (extracellular plus intracellular) and intracellular organisms in both animal models for both wild type and *ΔinvA *mutants was very low relative to the total number of organisms in the whole cecum including the cecal contents. Strikingly in chicks, wild type serotype Typhimurium ATCC14028 was associated with cecal tissue in higher numbers, although intracellular numbers of wild type organisms and *ΔinvA *organisms were similar (Figure 3A and 3C, Day 1, cell associated vs. intracellular). This data suggests that *ΔinvA *mutants were somewhat defective for attachment to the intestinal epithelium in the chick and this was not the case in infected mice.

**Figure 4:**
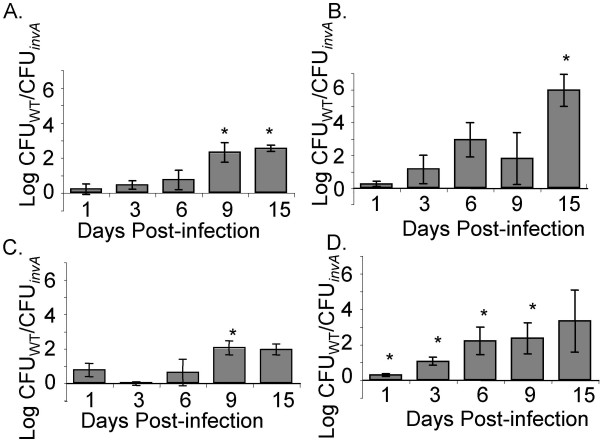
**Colonization of the whole cecum and cecal contents by ATCC14028 and Δ*invA *mutants**. Recovery of wild type serotype Typhimurium ATCC14028 and Δ*invA *mutant in the whole cecum (tissue + contents) of chicks (A) and mice (B) and in the isolated cecal contents of chicks (C) and mice (D) is shown as the mean log ratio of wild type versus mutant and error bars denote standard error. Asterisks indicate a significant difference relative to the ratio of wild type vs. mutant in the inoculum, at *P *< 0.05.

To further analyze the influence of the SPI-1 encoded TTSS-1 on colonization of the murine and chick intestine over time, we represented our data from competitive infections of wild type serotype Typhimurium ATCC14028 and our Δ*invA *mutant as the normalized log (wild type CFU/Δ*invA *mutant CFU) for each cecal compartment we analyzed in these experiments over 15 days of infection. We have noted above that in both mice and chicks the Δ*invA *mutant initially colonizes the whole cecum and cecal contents in numbers very similar to the wild type (Figure 3 and Figure 4A, B, C, and 4D at 1 day post-infection). However, the Δ*invA *mutant is found in statistically significantly lower numbers (*P *< 0.05) in the whole cecum and cecal contents of chicks primarily at later time points, on days 9 and 15 post infection (Figure 4A and 4C). At these time points the number of Δ*invA *organisms in the whole cecum (tissue + contents) and in isolated cecal contents fell dramatically (Figure 3), while the numbers of wild type organisms did not.

In our murine model, the level of Δ*invA *mutants was statistically significantly lower than the isogenic wild type in the whole cecum (tissue + contents) at 15 days post infection, and this difference was of greater magnitude in mice than in chicks (Figure 4B). In examining the isolated cecal contents from infected mice, we noted that the wild type organisms were present in higher numbers in the cecal contents throughout the duration of the infection (*P *< 0.05), albeit very subtly at early time points post infection (Figure 4D).

**Figure 5:**
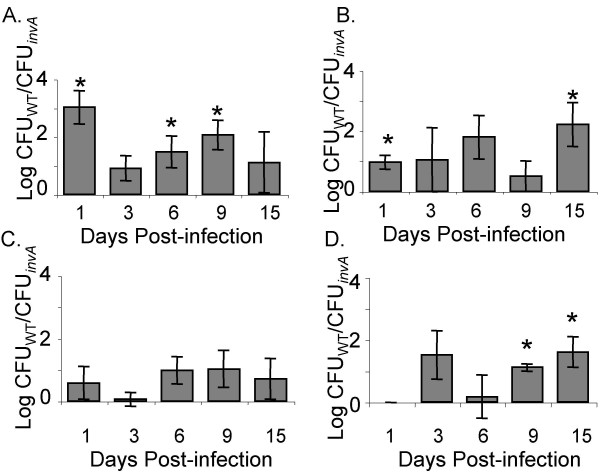
**Cell-associated and intracellular growth of ATCC14028 and Δ*invA *mutants**. Recovery of cell-associated (extracellular but firmly attached + intracellular) serotype Typhimurium and Δ*invA *mutant in the cecum of chicks (A) and mice (B) and intracellular wild type and Δ*invA *mutant in the cecum of chicks (C) and mice (D) is shown as the mean log ratio of wild type versus mutant. Error bars denote standard error and asterisks indicate a significant difference relative to the ratio of wild type vs. mutant in the inoculum at *P *< 0.05.

Analysis of the cell-associated component of serotype Typhimurium and Δ*invA *mutant growth in the cecum of infected chicks and mice included bacteria that were firmly attached to the epithelial cell wall and those that were intracellular (Figure 5). In both 1-week-old chicks and in mice there was a significant difference (*P *< 0.05) between wild type and mutant in the number of cell-associated organisms on day 1 post-infection (Figure 5A, B). This statistically significant difference appears to be driven by extracellularly attached bacteria, as the numbers of intracellular wild type and Δ*invA *mutant were very similar at this early time point post infection in both the chick and murine models we studied (Figure 5C and 5D, and Figure 3 all panels). In addition we show a significant difference (*P *< 0.05) in cell associated CFU between the two strains on days 6 and 9 in chicks (Figure 5A) that also appears to be driven by differences in extracellular organisms that are firmly attached to cecal tissue (Figure 5A and 5C). Finally, we noted that in mice wild type *S*. Typhimurium ATCC14028 was found in higher numbers in the cell associated fraction than our Δ*invA *mutants at very late time points post infection (15 days, Figure 5B). This defect of Δ*invA *mutants to colonize murine cecal tissue appears to be due, at least in part, to a reduced ability to become intracellular (Figure 5D, 9 and 15 days). Our observations that Δ*invA *mutants are poorly able to invade the epithelium are consistent with previously published findings [8].

### Fecal shedding of wild type versus Δ*invA*

**Figure 6:**
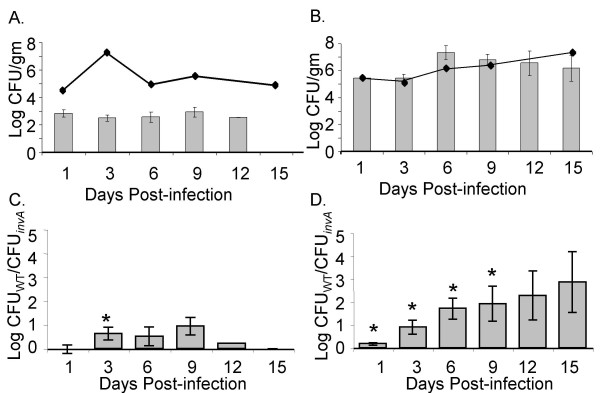
**Dynamics of *S*. Typhimurium fecal shedding in chicks and mice**. Recovery of wild type ATCC14028 in the feces of chicks (A) and mice (B) is shown as mean log CFU/g of feces. Lines represent the level of cecal colonization of each animal model. Recovery of wild type and Δ*invA *mutant in the feces of chicks (C) and mice (D) is shown as the mean log ratio of wild type versus mutant. Error bars indicate standard error, and asterisks indicate a significant difference relative to the ratio of wild type vs. mutant in the incoculum at *P *< 0.05.

We analyzed the level of fecal shedding of wild type serotype Typhimurium ATCC14028 and our Δ*invA *mutant, from both infected chicks and infected CBA/J mice. We show that the overall level of fecal shedding of wild type serotype Typhimurium is greater from infected mice than from infected chicks (Figure 6A, B). Furthermore, the level of fecal shedding of serotype Typhimurium in infected chicks does not accurately reflect the number of bacteria in the chick ceca (Figure 6A, line represents the level of cecal colonization). However, this is not the case in *Salmonella*-infected CBA/J mice, where the level of fecal shedding correlated very accurately with the level of cecal colonization (Figure 6B, line represents the level of cecal colonization).

We also compared the level of fecal shedding of our serotype Typhimurium wild type versus our Δ*invA *mutant from infected chicks and infected CBA/J mice (Figure 6C and 6D). In the feces of infected chicks, there was no difference in the levels of wild type serotype Typhimurium and Δ*invA *mutant shed in the feces throughout the infection, both strains were shed at low but identical levels from infected chicks (Figure 6C). In mice, wild type serotype Typhimurium had an advantage over Δ*invA *mutant at all time points with a statistically significant (*P *< 0.05) difference on days 1, 3, 6 and 9 post infection (Figure 6D).

## Discussion

*Salmonella enterica *serotype Typhimurium is a non-pathogenic commensal organism in chicks greater than 3 days of age and can colonize the intestinal tract subclinically for 8–9 weeks in >90% of birds after experimental infection [29]. This subclinical carriage has consequences for human health, as broilers are currently brought to slaughter at about 40 days of age, well within this period. Very little is known about the bacterial factors that are required for serotype Typhimurium to colonize and survive in the chick intestinal tract, and very few studies have been performed in chickens utilizing Typhimurium isolates that have been heavily studied in murine models and *in vitro*.

We investigated the distribution and dynamics of colonization of *Salmonella enterica *serotype Typhimurium ATCC14028 and an isogenic mutant in the *invA *locus (SPI-1), following oral inoculation in 1-week-old SPF White Leghorn chicks and *Salmonella*-resistant CBA/J mice. While several studies have examined serotype Typhimurium colonization of chicks [24,34-36], ours is the first study to directly compare colonization of these two model hosts with ATCC14028, one of the most highly studied Typhimurium isolates in both murine and *in vitro *cell culture systems. ATCC14028 has been studied intensively in murine and *in vitro *model systems for the last 20 years, and its complete genome sequence is currently being determined.

We show that 1-week old chicks infected with serotype Typhimurium ATCC14028 have very few organisms found in systemic organs and also show no disease or gross evidence of systemic effects (organ enlargement). In CBA/J mice, despite the absence of clinical disease, both the spleen and the liver became increasingly colonized throughout the infection and corresponding splenomegaly and hepatomegaly were apparent at 15 days post infection. CBA/J mice are resistant to the development of systemic disease after infection with serotype Typhimurium because they possess a wild type NRAMP1^G169 ^allele (Natural Resistance Associated Macrophage Protein 1, *SlC11a1*) that encodes an integral membrane protein that is a manganese transporter found on lysosomes [37-39]. Survival and growth within macrophages is critical to the virulence of *Salmonella *and NRAMP1 is responsible for restricting the growth of *Salmonellae *in this cell type [40,41]. It is possible that, despite this growth restriction, serotype Typhimurium may grow at a low level in systemic organs such as the spleen in CBA/J mice. This low level growth could partially account for the increasing colonization of systemic organs that we observed in our study.

We show that colonization of the intestine by serotype Typhimurium ATCC14028 also has a different dynamic in the 1-week old chick versus the *Salmonella *resistant-mouse model of infection. As previously reported, the cecum is a highly colonized site in both host species we studied [28,42-44]. In 1-week old chicks in our studies, the cecum is most highly colonized on day 3 post infection, and coincident with this the systemic organs are most highly colonized as well. In mice, cecal and systemic colonization both increase with time after infection. This slowly increasing systemic colonization and host response in murine models may be due to continuous re-seeding of systemic organs from highly colonized areas of the gut, including the cecum, via gut associated lymphoid tissues (GALT).

We have only recently begun to appreciate the complexity of, and the interaction of pathogens with the intestinal microflora of the host. Two studies have shown recently that serotype Typhimurium disrupts the intestinal microflora in murine models after experimental *Salmonella *infection, and promotes neutrophilic intestinal inflammation that slows the recovery of this microflora [45,46]. These mechanisms in the *Salmonella*-resistant mouse may participate to allow serotype Typhimurium to colonize the intestine for prolonged periods of time, and at increasing levels. Although 2-week old chickens have also been reported to have a heterophilic influx in the cecum of serotype Typhimurium infected animals that reaches a high level at 48 hours post infection, the effect of this inflammatory influx on the avian flora have not been studied, and the chick cecal epithelium remains intact despite the inflammatory response [47]. Future studies of both the murine and chick microflora are needed to determine the role of this important microbial community in the differential development of gastrointestinal disease caused by *Salmonella*, and on containing systemic spread of these organisms in different host species.

Within the cecum of infected chicks and mice in our experiments, bacteria were largely located in the cecal contents with a smaller number associated with the cecal epithelium. Intracellular localization in the cecum is not a prominent niche for chick or murine cecal colonization during gastrointestinal colonization, although intracellular localization of *Salmonella *in Peyer's patches and in solitary intestinal lymphoid tissue (SILT) of the small intestine is thought to be critical for disease, systemic spread and the development of intestinal pathology in the mouse [19,42,48]. Chicks have rare Peyer's patches in the ileum (reported to be 1–2) in 50% of chicks at 10 days of age [49] and additional lymphoid tissue at the tip of the cecum and in the cecal tonsil, although the distribution of SILT is unstudied in the chick [50]. In the chick, this different, and possibly lower, distribution of GALT in the small intestine may result in lower levels of colonization of the intestinal epithelium in the chick.

We investigated the importance of SPI-1, encoding the TTSS-1, in colonization of the cecum during serotype Typhimurium infection in White Leghorn chicks and *Salmonella*-resistant mice. The SPI-1 TTSS is important for colonization of mammalian epithelial cells [8,15,33,51,52], but the role of this important virulence factor in chickens is less clear. *Salmonella *pathogenicity island 1 (SPI-1) is not important for colonization of cecal contents in 1-day old Rhode Island Red chicks, at either 1,2, or 3 days post infection [34]. SPI-1 mutants colonize the cecal contents of 1-week old Rhode Island Red chicks normally at one day post infection but have reduced colonization of cecal contents over the longer term at 3,7, and 14 days post infection [34]. SPI-1 genes were not identified as important for infection during recent Signature Tagged Mutagenesis screening in 2-week-old SPF Light Sussex chicks at 4 days post infection [24]. Our results confirm these earlier findings in that Typhimurium ATCC14028 SPI-1 mutants colonize the cecum and cecal contents of chicks initially in numbers identical to the isogenic wild type. Our current analysis goes beyond previously published work to show that having an intact TTSS-1 in the chick, while having little influence on invasion of the cecal epithelium, seems to affect the extracellular association of serotype Typhimurium with the cecal epithelium. This finding in chicks is in contrast with previous studies utilizing cultured mammalian epithelial cells showing that Δ*invA *mutants are not deficient in extracellular attachment but are only deficient in their ability to invade cultured mammalian epithelial cells [8,33]. Our findings suggest that serotype Typhimurium is not likely to be dependent on the intracellular niche for replication in the chick cecum.

The results of our study indicate that SPI-1 is important for maintaining the levels of Typhimurium in the cecal contents in *Salmonella-*resistant CBA/J mice from the beginning of the infection, despite the fact that intracellular levels of Δ*invA *mutants in the cecal epithelium are similar to wild type until very late time points post infection. These findings may indicate that intracellular replication of serotype Typhimurium in the cecal epithelium is not the major determinant of the level of cecal colonization in the mouse. Perhaps continuous seeding of the cecum occurs from bacterial growing intracellularly in the distal ileum, a niche where Typhimurium is known to replicate in the GALT using SPI-1, contributes to maintaining the high level of colonization of the cecum in serotype Typhimurium infected mice.

Finally, we also studied the congruence between cecal colonization and fecal shedding of serotype Typhimurium ATCC14028 in both chicks and mice. The bacterial counts in the feces of chicks were consistently two orders of magnitude less than those seen in the chick cecum, although fecal shedding and cecal colonization in *Salmonella*-resistant mice are well correlated. Although sampling of cecal contents has been used previously to evaluate the level of serotype Typhimurium colonization, sampling cecal contents has not previously been compared to fecal shedding as a method for estimating *Salmonella *colonization of the gastrointestinal tract in chicks [22,47]. Evaluating fecal shedding of serotype Typhimurium is a common method used to determine extent of intestinal colonization during serotype Typhimurium persistence studies in murine models of infection, and our data indicate that this method does not accurately evaluate intestinal colonization in chicks. In addition, the bacterial numbers of serotype Typhimurium and Δ*invA *mutant in the feces of mice reflected those in the whole cecum, we show that this is not the case for chicks.

## Conclusion

We directly compare the colonization of a highly studied serotype Typhimurium isolate in two different animal models for the first time. This isolate, ATCC14028, is currently undergoing complete genome sequencing. We show that systemic colonization after oral infection in these two animal models is different, and has obvious systemic consequences in *Salmonella*-resistant murine models. Intestinal colonization also has different dynamics in both animal models, which may be partially responsible for influencing the presence or absence of increased systemic colonization. We also show that the level of intracellular colonization of the cecal epithelium in both animal models is very low, and the role of SPI-1 in colonization of the intestinal epithelium of the chick may be attachment rather than strictly invasion, although SPI-1 is known to be important for epithelial cell invasion *in vitro *and in other animal models. Finally, we show that fecal shedding of serotype Typhimurium in infected 1-week old White Leghorn chicks does not accurately reflect the level of intestinal colonization.

Placing our colonization and fecal shedding data from two different animal models in the context of complete genome sequence information may allow us to begin to determine the genetic basis for different levels of colonization in the murine and chick gastrointestinal tracts. Establishment of a chick model of colonization with serotype Typhimurium ATCC14028 also facilitates this goal.

## Methods

### Animals

One-week-old unsexed SPF chicks were obtained from Charles River SPAFAS (North Franklin, CT). They were housed in a poultry brooder (Alternative Design Manufacturing, Siloam Springs, AR) in groups of five or six with *ad libitum *access to tap water and irradiated lab chick diet (Harlan Teklad, Madison, WI). Brooder temperature was maintained at 32°C to 35°C. Eight-week-old female CBA/J mice were obtained from The Jackson Laboratory (Bar Harbor, Maine). Animals were housed in standard polycarbonate microisolator cages (Alternative Design Manufacturing, Siloam Springs, AR) containing aspen shredded bedding (Harlan Teklad, Madison, WI) in groups of three to four. Mice were fed a 4% standard rodent chow (Harlan Teklad, Madison, WI) with free access to food and water. All procedures described in this study were approved by the Texas A&M University Institutional Animal Care and Use Committee. Chicks were specific pathogen free for the following agents: avian adenovirus group I, avian adenovirus group II (HEV), avian adenovirus group III (EDS), avian encephalomyelitis, avian influenza (Type A), avian Reovirus, chick anemia virus, fowl pox, infectious bronchitis, infectious bursal disease type 1, infectious bursal disease type 2 infectious laryngotracheitis, Marek's disease (serotypes 1, 2, 3), *Mycoplasma gallisepticum*, *Mycoplasma synoviae*, Newcastle disease, reticuloendotheliosis, *Salmonella enterica *subspecies Enterica serotypes Enteritidis, Pullorum, Gallinarum, and other *Salmonella *serotypes.

### Bacterial strains and growth conditions

The strains used in this study are summarized in Table 1. A spontaneously nalidixic acid resistant (Nal^r^) strain of *Salmonella enterica *serovar Typhimurium ATCC14028s, HA420, which is virulent and persistent in murine models, was used as our wild type. Strains bearing a mutation of the *phoN *gene (ATCC14028s Δ*phoN*::Kan, HA431 and ATCC14028s Δ*phoN*::Cm, HA530) were constructed using the lambda red recombinase method [53]. *phoN+ *and *phoN- *strains can be distinguished by blue-white selection on 5-bromo-4-chloro-3-indolyl phosphate (XP) containing media. *phoN- *strains form white colonies while *phoN+ *strains appear blue. Mutations in *phoN *do not affect the ability of serotype Typhimurium to colonize organs or persist in the intestinal tract in murine or chick models [54](and data not shown). The use of multiple antibiotic cassettes in generation of the mutant strains served as an additional tool for identification of wild type versus mutant colonies on selective media.

**Table 1:** Serotype Typhimurium strains used in this study.

Strain	Genotype	Reference
HA420	ATCC14028s Nal^r^, wild type	[55]
HA431	ATCC14028sΔ*phoN*::Kan, wild type	This study
HA530	ATCC14028sΔ*phoN*::Cm, wild type	This study
HA460	ATCC14028sΔ*invA*::Kan	This study

*Salmonellae *were routinely cultivated in Luria-Bertani (LB) broth at 37°C or 42°C, on LB plates and on Brilliant Green plates supplemented with the appropriate antibiotics. Bacterial cultures to be used to infect chicks or mice were grown overnight to stationary phase in LB broth supplemented with the appropriate antibiotics at 37°C or 42°C with aeration. Antibiotics and other supplements were used at the following concentrations: 20 mg/L XP and 100 mg/L nalidixic acid (Nal) or Brilliant Green agar containing 100 mg/L Nal and either 20 mg/L chloramphenicol (Cm) or 50 mg/L kanamycin (Kan).

### P22 transduction

Prior to their use in experiments involving chicks and mice, all mutants were transferred to a clean genetic background by P22 transduction. Briefly, the mutant strains were grown overnight as described above. Lysates were prepared using serial dilutions of P22 (10^-1 ^to 10^-4^) and 100 μl of the overnight culture. Wild type HA420 was grown overnight and was used as the recipient strain. Recipient strain and lysate were mixed and incubated with LB and 10 mM EGTA to inhibit lysis. The mixture was plated on LB with EGTA and the appropriate antibiotic marker and incubated as previously described. Colonies were picked for streaking on EBU plates with antibiotic to check for loss of phage. Once purification was determined, mutants were stored in 30% glycerol at -80°C until use.

### Single infection

Thirty-six 1-week-old SPF White Leghorn chicks in groups of 6 were inoculated orally with 6 × 10^8 ^CFU of HA420 in 0.1 ml LB broth. Twenty-four control chicks in groups of 4 were inoculated orally with 0.1 ml sterile LB broth. Control birds were housed separately from infected birds to minimize cross contamination. Six birds from the infected group and 4 from the control group were euthanized by CO_2 _asphyxiation on days 1, 3, 6, 9 and 15 post-infection. Liver, spleen, sections of large and small intestine and one cecal arm (cecum plus contents) were collected. A portion of each organ collected was weighed and placed in 3 ml sterile PBS for bacteriology. Following homogenization, samples were serially diluted and plated on LB agar containing Nal and XP for determination of colony forming units (CFU).

Twelve female CBA/J mice were inoculated orally with 1.5 × 10^10 ^CFU of HA420 in 0.1 ml LB broth and 12 control mice were inoculated orally with 0.1 ml sterile LB broth. Liver, spleen, cecum, and sections of small and large intestine were collected on days 1, 3, 9 and 15 days post infection. Samples were processed as described for chicks to enumerate CFU.

### Competitive infection

Twenty-seven 1-week-old SPF White Leghorn chicks were inoculated orally with 2 × 10^8 ^CFU of a 1:1 mixture of wild type HA530 and mutant HA460 in a volume of 0.1 ml in PBS. The inoculum was serially diluted for determination of CFU and the exact input ratio. Fecal samples were collected by cloacal swab on days 1, 3, 6, 9, 12 and 15 post-infection. Samples were weighed and serially diluted. Double plating was performed on Brilliant Green agar containing Nal and Kan and Brilliant Green agar containing Nal and Cm for determination of CFU of wild type versus mutant strain.

Groups of five or six chicks were euthanized on days 1, 3, 6, 9, and 15 post infection and the cecum was collected. One cecal arm (cecum plus contents) from each chick was placed in 3 ml sterile PBS. Samples were weighed, homogenized, serially diluted and plated as described for fecal samples. In order to determine how many CFU were luminal, cell associated and intracellular, the contents from the second cecal arm for each chick was emptied into 3 ml sterile PBS and the wall was split into two sections. One section of the cecal wall was placed into 3 ml sterile PBS and the other was incubated in 3 ml sterile PBS containing 100 μg/ml gentamicin at 37°C for 90 minutes. Following this treatment, gentamicin was removed by washing the sections three times with sterile PBS. Cecal wall sections were homogenized and serial dilutions and plating of both wall sections and contents was performed as described above to determine CFU.

Twenty 8-week-old female CBA/J mice were inoculated orally with 1.4 × 10^9 ^CFU of a 1:1 mixture of wild type strain HA431 and mutant strain HA460 in 0.1 ml sterile PBS. Three fecal pellets (approximately 100 mg) were collected from each mouse on days 1, 3, 6, 9, and 15 post infection. Samples were weighed, serially diluted and plated. Four mice were euthanized on days 1, 3, 6, 9 and 15 post infection and the cecum was collected. Half of the cecum (cecum plus contents) from each mouse was weighed and placed in 3 ml sterile PBS for plating as described for the fecal samples. In order to determine luminal, cell associated and intracellular CFU the contents from the remaining half of the cecum was placed in 3 ml sterile PBS and the remaining cecal wall was split into two sections for processing as described previously. All murine samples were plated on LB agar containing Nal and XP for determination of CFU of wild type versus mutant strain.

### Statistical analysis

A two-tailed Student's *t *test for independent samples was used to compare the differences in liver and spleen weights between infected and control groups of chicks and mice. *P *values of < 0.05 were considered statistically significant. Data obtained from competitive infection experiments were calculated as a mean log ratio of wild type versus mutant normalized to the input ratio. Statistical significance was determined using a two-tailed Student's *t *test and *P *values of < 0.05 were considered statistically significant (SPSS software, SPSS, Inc., Chicago, IL).

## Authors' contributions

LMB and HLAP conceived and designed the experiments. CPS and LMB performed the experiments. CPS, LMB and HLAP analyzed the data and interpreted the results. CPS and HLAP wrote the paper.
